# A 5-year longitudinal analysis of modifiable predictors for outdoor play and screen-time of 2- to 5-year-olds

**DOI:** 10.1186/s12966-016-0422-6

**Published:** 2016-08-26

**Authors:** Huilan Xu, Li Ming Wen, Louise L Hardy, Chris Rissel

**Affiliations:** 1 Sydney School of Public Health, University of Sydney, Sydney, NSW 2006 Australia; 2 Health Promotion Unit, Sydney Local Health District, Level 9, King George V Building, Missenden Road, Camperdown, NSW 2050 Australia; 3 Prevention Research Collaboration, Sydney School of Public Health, University of Sydney, Sydney, NSW 2006 Australia

**Keywords:** Physical activity, Outdoor play, Screen-time, Predictor

## Abstract

**Background:**

Early childhood is a critical time for establishing physical activity and sedentary behaviours. Identifying modifiable predictors of physical activity and sedentary behaviours in the early life stages can inform the development of early intervention programs. The aim of this study was to identify modifiable predictors of outdoor play (a proxy of physical activity) and screen-time in 2- to 5-year-olds.

**Methods:**

A longitudinal data analysis was conducted using 5-year follow-up data from the Healthy Beginnings Trial undertaken in Sydney, Australia from 2007 to 2013. A total of 667 pregnant women were recruited for the study. Information on mothers’ demographics, physical activity, screen-time, knowledge of child development, and awareness of childhood obesity during pregnancy (at baseline); children’s tummy time (a colloquial term describing the time when a baby is placed on his or her stomach while awake and supervised) at 6 months old and screen-time at 1 year old was collected via interviews with participating mothers as potential modifiable predictors. Main outcomes were children’s outdoor playtime and screen-time at ages 2, 3.5, and 5 years. Mixed linear and logistic regression models were built to determine these modifiable predictors.

**Results:**

Mothers’ screen-time during pregnancy (β = 2.1, 95 % CI 0.17–4.12; *P* = 0.030) and children’s daily screen-time at age 1 year (β = 15.2, 95 % CI 7.28–23.11; *P* < 0.0001) predicted children’s daily screen-time across ages 2 to 5 years after controlling for confounding factors. Practising tummy time daily (β = 13.4, 95 % CI 1.26–25.52; *P* = 0.030), mother’s physical activity level (β = 3.9, 95 % CI 0.46–7.28; *P* = 0.026), and having been informed about playing with child at baseline (β = 11.6, 95 % CI 1.56–21.54; *P* = 0.023) predicted children’s outdoor playtime across ages 2 to 5 years.

**Conclusions:**

Mothers played an important role in their children’s outdoor play and screen-time in the first years of live. Children’s early exposure to screen devices could be associated with their later screen-time. Early interventions to improve young children’s physical activity and sedentary behaviour should focus on improving pregnant women’s physical activity, awareness of playing with their child, reducing their own screen-time as well as practicing daily tummy time for infants after giving birth.

**Trial registration:**

The Healthy Beginnings Trial is registered with the Australian Clinical Trial Registry (ACTRNO12607000168459). Registered 13 March 2007. Prospectively registered.

## Background

Physical activity and sedentary behaviours of young children have been gaining public health attention with increasing early onset and high prevalence of childhood overweight and obesity [1, 2]. Indeed, recommendations for how long children should spend in physical activity and sedentary behaviours each day have been developed in many countries to guide parents and carers of young children. The National Association for Sport and Physical Education in the U.S.A recommends that 3- to 5-year-olds should accumulate at least 60 min daily of structured physical activity; engage in at least 60 min daily unstructured physical activity; and should not be sedentary for more than 60 min at a time except when sleeping [3]. In Australia, the Department of Health recommends that 2- to 5-year-olds should be physically active for at least 3 h per day (accumulated throughout the day including light-, moderate-, and vigorous-intensity physical activity); spend less than one hour per day watching television and using other electronic media; and not be sedentary, restrained, or kept inactive for more than one hour at a time with the exception of sleeping [4]. The United Kingdom and Canada endorsed similar guidelines of physical activity and sedentary behaviour for young children [5–7].

Many people believe that young children are naturally physically active [8]. However, many 3- to 5-year-olds are not as physically active as they need to be for good health, with one-in-twenty and less than one-in-seven Australian preschool children meeting physical activity and screen-time recommendations respectively [9]. Previous systematic reviews examining objectively measured physical activity within child-care centers also concluded that physical activity levels of preschool children are typically very low and levels of sedentary behaviour are high [10, 11]. Ensuring adequate physical activity and preventing excessive sedentary behaviour, in particular, screen-time, not only provides benefits for weight control in children but also benefits their physical development and psychological well-being [12–14]. Therefore, interventions in improving children’s physical activity behavior during their early years will be important in preventing childhood overweight and obesity and improving children’s well-being [15].

In recent years, several systematic reviews have summarized the factors associated with physical activity and sedentary behaviours in young children [16–19]. Most studies included in systematic reviews were cross-sectional studies and identified correlates of physical activity and sedentary behaviours, many of which are not modifiable, such as children’s sex, age, ethnicity, socioeconomic status. Longitudinal studies that examine predictors of physical activity and sedentary behaviours in young children are scarce. Although identifying demographic and socioeconomic factors that are associated with physical activity and sedentary behaviours are important for targeted population interventions, identifying modifiable predictors can lead to more effective interventions. Among young children, physical activity occurs predominantly during active play. Outdoor playtime is often used as a proxy of physical activity of young children [20]. Therefore, the present study aimed to determine modifiable factors in early life that predict outdoor play and screen-time in 2- to 5-year-olds.

## Methods

### Study design

A longitudinal data analysis was conducted using data extracted from the Healthy Beginning Trial (HBT), which was a 5-year randomised controlled trial undertaken in south-western Sydney, Australia, during 2007–2013. The details of the research protocol and the main outcomes of the HBT have been reported elsewhere [21, 22]. Briefly, the HBT assess the effectiveness of a staged home based early intervention in reducing early childhood obesity. The intervention comprises eight home visits from a specially trained community nurse promoting healthy feeding and physical activity. The intervention started from the gestation age of 30–36 weeks to children reaching 2 years old. Five-year follow up data were obtained from mothers at the late pregnancy to children’s ages at 6 months, 1, 2, 3.5, and 5 years old. The study was approved by the Ethics Review Committee of Sydney South West Area Health Service (Royal Prince Alfred Hospital Zone, X10-0312 & HREC/10/RPAH/546). Since the HBT was a randomised controlled trial the group allocation (intervention or control) was taken into account in the data analysis.

### Study participants

A total of 667 first-time mothers at 24–34 weeks of pregnancy were recruited from antenatal clinics at Liverpool and Campbelltown Hospitals, located in south-western Sydney, Australia. The analysis included 497, 415, and 369 mother-child dyads that were retained at ages 2, 3.5, and 5 years.

### Data collection and measures

Face-to-face interviews with each mother at their home were conducted by trained research nurses at baseline (30 to 36 weeks of pregnancy), 1, 2, 3.5, and 5 years follow-up. A telephone survey was conducted when each child was 6 months old. Potential modifiable predictors were assessed at baseline, 6 months, and 1 year follow-up.

#### Children’s outdoor play and screen-time

Children’s outdoor playtime was measured at ages 2, 3.5, and 5 years. To assess outdoor playtime, the mother was asked how much time her child spent playing outdoors on a typical weekday and on a typical weekend day with validated survey questions [20]. The mean outdoor playtime per day was calculated as (hours/weekday × 5 + hours/weekend day × 2)/7. Outdoor playtime was then categorised into ‘<2 h/day’ and ‘≥2 h/day’ (based on the median outdoor playtime around 2.3 h/day).

Children’s screen-time was measured at ages 1, 2, 3.5, and 5 years. Survey questions from a national-wide study called Growing Up in Australia: The Longitudinal Study of Australian Children were used to assess screen-time [23]. The mother was asked to provide the total time her child spent on each of the activities including (i) watching TV programmes; (ii) watching DVDs or videos; (iii) using a computer; and (iv) playing with an electronic game system from Monday to Friday and also on weekends [23]. Screen-time was summed and the mean screen-time (hours per day) was calculated. Screen-time was then categorised into ‘<1 h/day’ and ‘≥1 h/day’ based on the screen-time recommendation for children aged 2–5 years [4]. For full-time employed mothers, they were asked to estimate their child’s outdoor playtime and screen-time based on the time when they were with the child.

#### Mothers’ physical activity and screen-time

Questions from the Active Australia Survey questionnaire 2003 [24] were used to assess mothers physical activity level before pregnancy, at 2, 3.5, and 5 years follow-up. Mother’s total physical activity time was calculated as hours per day and further categorised into ‘<150 min/week’ and ‘≥150 min/week’ based on the recommendation of physical activity for adults [25].

Mother’s screen-time was assessed by a question ‘Currently, on average, how many hours per day or per week do you spend sitting watching TV, videos, DVDs, playing computer or video games, or surfing the Internet for pleasure?’ It was reported by mothers at baseline, 2, 3.5 and 5 years. Screen-time was calculated as hours per day. Baseline screen-time was further categorised as ‘<3 h/day’ and ‘≥3 h/day’ based on the mean value of 3.1 h/day from the study participants at baseline.

#### Mothers’ knowledge and awareness

Mothers’ knowledge of child development at baseline was assessed by three questions including 1) How informed do you feel about child development e.g. age a child typically crawls, walks, runs? 2) How informed do you feel about playing with children? 3) How able do you feel you are to give your child activities that will keep them occupied while you are doing other things? The response was chosen from a 4-point Likert-type scale (very informed/able, somewhat informed/able, a little informed/able, and not at all informed/able). The responses were categorized into ‘yes’ or ‘no’, with ‘yes’ referring to ‘very informed/able’.

Mothers’ awareness of childhood obesity at baseline was assessed by a question ‘How worried should adults be about their children being overweight or obese?’ The response was chosen from a 4-point Likert-type scale (Extremely worried, Very worried, A little worried, Not at all worried). The responses were categorized into ‘yes’ or ‘no’, with ‘yes’ referring to ‘Extremely/very worried’.

#### Tummy time and playgroup

Children’s tummy time is when a baby is placed on his or her stomach while awake and supervised. Children’s tummy time frequency and starting time were reported by mothers when children were 6 months old. Tummy time frequency was assessed by a question ‘How often does your child spend time on their tummy when they are awake?’ [26] Response options were ‘Not at all’, ‘1–2 days a week’, ‘3–4 days a week’, ‘5–6 days a week’, ‘Daily’. The responses were categorized into ‘daily’ and ‘not daily’. Age of starting tummy time was assessed by a question ‘At what age did your child start spending time on [his/her] tummy when [he/she] was awake?’ It was calculated as days of age and then categorised into within or after 1 month (30 days) of birth according to the mean starting tummy time 30 days.

Attendance of childhood program or activity was measured at age 1 year by a question ‘Does your baby currently attend any play group or other early childhood program or activity?’ The responses were ‘yes’ or ‘no’.

#### Mothers’ demographics

Mothers’ demographic and socioeconomic information were collected at baseline, 1, 2, 3.5, and 5 years using questions from the NSW Child Health Survey 2001 [26]. All mothers’ demographic and socioeconomic information were categorized into groups (see Table 1).

**Table 1:** Baseline characters of mothers and children at baseline, 2, 3.5, and 5 years

Baseline demographics	Baseline	2 years	3.5 years	5 years
	n (%)	n (%)	n (%)	n (%)
Mother’s age (years)^a^				
16–24	279 (42)	185 (37)	140 (34)	117 (32)
25–29	226 (34)	176 (36)	153 (37)	139 (38)
≥ 30	162 (24)	136 (27)	122 (29)	113 (30)
Mother’s country of birth				
Other	237 (36)	175 (35)	151 (36)	130 (35)
Australia	429 (64)	321 (65)	263 (64)	238 (65)
Mother’s education status^b^				
Completed primary school/School Certificate	137 (21)	82 (16)	57 (14)	46 (12)
HSC to TAFE certificate or diploma	364 (55)	280 (57)	234 (56)	208 (57)
University	163 (24)	133 (27)	123 (30)	114 (31)
Mother’s employment status^a^				
Employed	363 (54)	295 (59)	261 (63)	238 (65)
Other	303 (46)	201 (41)	153 (37)	131 (35)
Mother’s marital status^a^				
Married/partner	584 (88)	452 (91)	382 (92)	343 (93)
Other	81 (12)	45 (9)	33 (8)	26 (7)
Annual household income ($AUD)^a^				
< 40,000	208 (31)	130 (26)	96 (23)	82 (22)
40,000–< 80,000	215 (32)	159 (32)	130 (31)	114 (31)
≥ 80,000	244 (37)	208 (42)	189 (46)	173 (47)
Child sex				
Girls	333 (50)	249 (50)	202 (49)	183 (50)
Boys	330 (50)	248 (50)	213 (51)	186 (50)

Note: sample size is not always 667, 497, 415, and 369 at baseline, 2, 3.5, and 5 years due to missing values

^a^Compare to baseline distribution, the distribution of the baseline variable at ages 3.5 and 5 years were significantly different

^b^Compare to baseline distribution, the distribution of the baseline variable at ages 2, 3.5 and 5 years were significantly different

#### Other covariates

Children’s night sleep duration, child-care/school attendance, TV time and programme viewing rules, whether TV is on all the time and during meals were measured at ages 2, 3.5 and 5 years.

Children’s night sleep duration (hours per night) was measured using questionnaire items from the Prevention of Overweight in Infancy study developed from the consensus opinion of the researchers [27]. Child-care/school attendance was measured using questions from the state population health survey [26], the mother was asked “Is your child currently having any type of formal or informal child-care on a regular basis?” and “Does your child attend school yet?” Response options were “yes” or “no”. To assess TV rules for children, the mother was asked ‘Are there rules about what TV programmes your child can watch?’ and ‘Are there rules about how many hours of TV your child can watch?’ The mother was also asked ‘How often is a TV on when no one is watching?’ and ‘How often is a TV on during meals?’ Response options were ‘always’, ‘often’, ‘sometimes’, ‘rarely’ or ‘never’ [23]. The responses were regrouped as ‘yes’ or ‘no’ with ‘yes’ referring to ‘always’, ‘often’ or ‘sometimes’.

### Data analysis

Statistical analyses were carried out using Stata 13 [28]. Mean and standard deviation or number and percentage were reported to summarise children’s outdoor play and screen-time, mothers’ demographics and other study factors. Children’s outdoor play and screen-time were analysed as both continuous and binary outcome variables.

Considering the longitudinal design, random-intercept mixed models were built to take into account correlations between repeated measures. Also by building mixed models, to some extent, missing data can be implicitly imputed. Therefore, participants with partial missing data were still able to contribute information. Since children’s outdoor playtime and screen-time were included as both continuous and dichotomous outcomes, mixed linear and logistic models were built respectively. In order to examine whether predictors varied on weekday and weekend day, also overcome the limitation that working mothers and mothers whose children attending child-care might underestimated or overestimate children’s outdoor play and screen-time on weekday, mixed models were built for outdoor play or screen-time on weekdays, weekend days, and overall daily respectively.

Two steps were taken to identify predictors of outdoor play and screen-time. First, the relationship between each potential predictor or confounder (such as demographics, correlates etc.) and children’s outdoor playtime and screen-time was examined by including each potential predictor or confounder in a mixed linear or logistic model that adjusted for time and quadratic slopes for time. The reason for including quadratic slopes for time was to improve model fit. Second, all variables significant in the first analysis with *P* < 0.25 were entered into a multivariable mixed model. All multiple mixed models were adjusted for allocation of intervention to control the intervention effect, time and quadratic slopes for time. The least significant variables were progressively dropped until only those with *P* < 0.05 remained. Child-care attendance, mothers’ employment status at 2, 3.5, and 5 years, variables that significantly predicted outdoor play and screen-time on either weekdays or weekend-days were also remained in models. Subsequently, the variables which were not included in the model were given an extra chance to enter the final model one by one to see whether they were predictors or confounders. Interaction between time and the potential predictors and confounding factors were also included in all models to test whether their effect varied across three time points. The interaction was excluded from the final model if the interaction was not statistically significant (*P* > 0.05). Visual inspection of residual plots did not reveal any obvious deviations from linearity or normality, indicating that a random-intercept mixed linear model was appropriate.

## Results

There was no significant difference regarding mothers’ country of birth and children’s sex among mother-child dyads that were retained at ages 2, 3.5, and 5 years. Mothers lost-to-follow-up were typically young, unmarried, lower educated, unemployed, and had lower household income (see Table 1). The main study factors are summarised in Table 2 and show that mothers’ mean screen-time was 3.12 (SD 2.48) hours per day with nearly half of them having ≥3 h screen-time per day at baseline. Mothers’ mean physical activity before pregnant was 1.16 (SD 1.44) hours per week with 71 % mothers met physical activity recommendation. Most children practiced tummy time daily (79 %) and started tummy time (71 %) within 1 month of birth. The mean children’s screen-time at 1 year was 0.64 h per day (SD 0.82) with 74 % children met screen-time recommendation. Study outcomes were summarised in Table 3. Across ages 2 to 5 years, both children’s outdoor playtime (from 2.28 to 2.64 h per day) and screen-time (from 1.37 to 2.25 h per day) significantly increased.

**Table 2:** Main study factors

	Mean (SD)	n (%)
Baseline variables		
Mother’s screen-time	3.12 (2.48)	
< 3 h/day		347 (52)
≥ 3 h/day		318 (48)
Mother’s PA time	1.16 (1.44)	
< 150 min/week		191 (29)
≥ 150 min/week		476 (71)
Informed about playing with child		
No		336 (51)
Yes		329 (49)
Informed about child development		
No		538 (81)
Yes		127 (19)
Able to give child activities to keep them occupied		
No		332 (50)
Yes		333 (50)
Awareness of childhood obesity		
No		142 (21)
Yes		523 (79)
6 months variables		
Start tummy time		
After 1 month of birth		158 (29)
Within 1 month of birth		392 (71)
Tummy time frequency		
Not daily		115 (21)
Daily		442 (79)
1 year variables		
Child screen-time (hours/day)	0.64 (0.82)	
≥ 1 h/day		93 (26)
< 1 h/day		259 (74)
Play group attendance		
No		359 (68)
Yes		166 (32)

Note: sample size is not always 667, 561, and 527at baseline, 6 months, and 1 year due to missing values

**Table 3:** Children’s screen-time and outdoor playtime at 2, 3.5, and 5 years

Outcomes	2 years	3.5 years	5 years
	n (%)	n (%)	n (%)
Child screen-time			
≥ 1 h/day	310 (64)	378 (91)	333 (90)
< 1 h/day	175 (36)	37 (9)	36 (10)
Child outdoor playtime			
< 2 h/day	181 (37)	135 (33)	120 (33)
≥ 2 h/day	305 (63)	379 (67)	246 (67)
	Mean (SD)	Mean (SD)	Mean (SD)
Child screen-time (hours/day)	1.37 (1.02)	2.48 (1.49)	2.25 (1.27)
Child outdoor playtime (hours/day)	2.28 (1.21)	2.48 (1.28)	2.64 (1.37)

Note: sample size is not always 497, 415, and 369 at 2, 3.5, and 5 years due to missing values

### Predictors of children’s outdoor play and screen-time across ages 2 to 5 years

The group allocation was not significantly associated with either children’s outdoor playtime or screen-time. Results of multiple mixed linear models are shown in Table 4. Mothers’ daily screen-time at baseline and children’s daily screen-time at 1 year were positively associated with children’s screen-time across ages 2 to 5 years after adjusting for time, time^2^, intervention allocation, mothers’ country of birth, children’s night sleep duration, child-care attendance, TV is on all the time, TV time rules, mothers’ employment status and screen-time at 2, 3.5, and 5 years. Each one hour increase in mothers’ daily screen-time at baseline was associated with 2 min more children’s daily screen-time (95 % CI 0.17–4.12) and screen-time on a weekday (95 % CI 0.26–4.41). Each one hour increase in children’s daily screen-time at 1 year was associated with 15 min (95 % CI 6.21–22.90) and 18 min (95 % CI 6.40–28.83) more screen-time on a weekday and weekend day respectively. Compared with children whose mothers had less than 3 h screen-time per day at baseline, children whose mothers had ≥3 h daily screen-time at baseline had 12 min (95 % CI 1.93–21.74) and 15 min (95 % CI 2.27–27.60) more screen-time on a weekday and weekend day respectively.

**Table 4:** Predictors of children’s screen-time and outdoor play time at 2, 3.5 and 5 years

Variables	Children’s screen-time (minutes/day)^a^		
	Week day	Weekend day	Daily
	β (95 % CI)	β (95 % CI)	β (95 % CI)
Mothers’ baseline screen-time (hours/day)	2.3 (0.26 to 4.41)	1.7 (-1.02 to 4.34)	2.1 (0.17 to 4.12)
Children’s screen-time at 1 year (hours/day)	14.6 (6.21 to 22.90)	17.6 (6.40 to 28.83)	15.2 (7.28 to 23.11)
Mothers’ baseline screen-time			
< 3 h/day	_	_	_
≥ 3 h/day	11.8 (1.93 to 21.74)	14.9 (2.27 to 27.60)	12.9 (3.43 to 22.3)
	Children’s outdoor playtime (minutes/day)^b^		
Mothers’ baseline PA (hours/day)	2.7 (-0.70 to 6.18)	6.5 (2.06 to 11.04)	3.9 (0.46 to 7.28)
Baseline informed about playing with children			
No	_	_	_
Yes	9.4 (-0.65 to 19.47)	14.9 (1.75 to 28.05)	11.6 (1.56 to 21.54)
Tummy time frequency			
Not daily	_	_	_
Daily	12.6 (0.37 to 24.81)	16.6 (0.60 to 32.53)	13.4 (1.26 to 25.52)
Mothers’ baseline PA			
< 150 min/week	_	_	_
≥ 150 min/week	−0.8 (-11.78 to 10.13)	5.3 (-9.15 to 19.67)	0.37 (-10.53 to 11.28)

*PA* physical activity

^a^All models are adjusted for time, time2, intervention allocation, mothers’ country of birth; children’s night sleep duration, child-care attendance, TV time rules, TV is on all the time, mothers’ employment status and screen-time at 2, 3.5, and 5 years

^b^All models are adjusted for time, time^2^, intervention allocation, child sex, mothers’ country of birth and education status at baseline; child-care attendance, mothers’ employment status, TV time rules, interaction of TV time rules and time at 2, 3.5 and 5 years

Mothers’ physical activity level and being informed about playing with children at baseline and children’s tummy time frequency were positively associated with children’s outdoor playtime across ages 2 to 5 years after adjusting for time, time^2^, intervention allocation, child sex, mothers’ country of birth and education status at baseline, child-care attendance, mother’s employment status, TV time rules, interaction of TV time rules and time at 2, 3.5 and 5 years. Each one hour increase in mother’s physical activity time before pregnancy was associated with 6 min (95 % CI 2.06–11.04) more children’s outdoor playtime on a weekend day and 4 min (95 % CI 0.46–7.28) daily outdoor playtime. Children whose mothers having been informed about playing with children at baseline had 12 min (95 % CI 1.56–21.54) more daily outdoor playtime and 15 min (95 % CI 1.75–28.05) more outdoor playtime on a weekend day.

Children having tummy time daily had 13 (95 % CI 0.37–24.81) and 17 min (95 % CI 0.60–32.53) more outdoor playtime on a weekday and weekend day respectively. Whether children started tummy time within one month of birth was not associated with children’s outdoor playtime. Whether mothers met the physical activity recommendation and whether children attended play group or other early childhood program were not associated with children’s outdoor playtime. Mothers’ knowledge of child development, belief of being able to give child activities, and high awareness of childhood obesity were not associated with either outdoor play or screen-time.

When examining predictors of children meeting the screen-time recommendation across ages 2 to 5 years, only children’s daily screen-time at age 1 year was a significant predictor. Children who had longer screen-time at age 1 year were less likely to meet the screen-time recommendation at ages 2 to 5 years with adjusted odds ratio (AOR) 0.55 (95 % CI 0.34–0.90) for daily screen-time recommendation after adjusting for potential confounders listed earlier.

Children whose mothers having been well informed about playing with children at baseline were more likely to play outdoor ≥ 2 h per day on a weekend day with AOR 1.65 (95 % CI 1.06–2.55); children who started tummy time within 1 month of birth were more likely to play outdoor ≥ 2 h per day on a weekday with AOR 1.44 (95 % CI 1.00–2.08) after controlling for confounding factors. Mothers’ physical activity time at baseline and children’s tummy time frequency were not predictors of whether children played outdoor ≥ 2 h per day.

### Correlates of children’s outdoor play and screen-time across ages 2 to 5 years

Multiple mixed linear models also showed that children with Australia born and employed mother had around 16 min (95 % CI 6.2–25.8) and 14 min (95 % CI 5.0–23.2) less screen-time per day; children who had longer night sleep duration, attended child-care, and had TV time rules had 6 min (95 % CI 1.6–10.2), 11 min (95 % CI -0.6–22.5), 9 min (95 % CI 0.8–18.1) less screen-time per day respectively; children whose mothers had lower than mean screen-time (2 h per day) at 2, 3.5, and 5 years had 19 min (95 % CI 10.2–27.6) less screen-time per day; children from family that TV is on all the time and TV is on during meals had 20 min (95 % CI 10.8–28.9) and 23 min (95 % CI 14.5–31.2) more screen-time per day.

Boys were more active than girls accumulating an additional 16 min (95 % CI 5.6–25.3) more outdoor playtime per day. Children whose mothers were Australian born had 25 min (95 % CI 14.3–36.2) more outdoor playtime per day. Children from families carrying TV time rules had 22 min (95 % CI 8.8–35.5) more outdoor playtime per day at age 2 years but not at ages 3.5 and 5 years.

## Discussion

To our knowledge, this was the first study identifying modifiable predictors of outdoor play and screen-time of young children using 5-year longitudinal data. We found that mothers’ own screen-time at baseline and children’s daily screen-time at age 1 year predicted children’s daily, weekday, and weekend day screen-time across ages 2 to 5 years; practising tummy time daily predicted children’s daily, weekday, and weekend day outdoor playtime across ages 2 to 5 years; mother’s baseline daily physical activity time and having been well informed about playing with children significantly predicted children’s daily and weekend day outdoor playtime. Although the effect sizes found in this study were relatively small, the findings could potentially have public health significance at a population level. The findings reinforce that mothers own physical activity and screen-time behaviours are important to their children’s physical activity and screen-time in the first 5 years of live; and children’s early exposure to screen devices can lead to longer screen-time at ages 2 to 5 years. The findings also suggested that young children’s outdoor play and screen-time were influenced by different factors.

Mothers’ screen-time and the home screen environment (‘TV is on all the time’ and ‘TV is on during meal’) at 2, 3.5 and 5 years were significantly associated with children’s screen-time on both weekdays and weekend days across ages 2 to 5 years after adjusting for mother’s baseline screen-time and other confounders. We found that the association between mothers’ and children’s screen-time are comparable with the findings from previous systematic reviews [18, 19, 29] that mothers’ screen-time serves as both a predictor and correlate of young children’s screen-time. This highlights the significant impact of mothers’ own screen viewing behaviour and practice on their children’s screen-time. Children from families with TV time rules, Australia born mothers and mothers who were employed had significantly less screen-time on weekdays but not on weekend days. This finding may indicate that independent of differences in culture, employment status, and family rules, most mothers would consider the weekend is time for entertainment, and therefore, allow children more screen-time.

Mothers’ pre-pregnancy physical activity level and being informed about playing with their child had a significant impact on children’s outdoor play on weekend days but not on weekdays. This may reflect families having more free time on the weekend than weekdays and those mothers tend to arrange more outdoor play for their children or themselves as well.

The timing of when tummy time was introduced was not significantly associated with children’s outdoor play while practising tummy time daily were associated with outdoor playtime on both weekdays and weekend days. It might suggest that frequency of having tummy time is more important in relation to young children’s physical activity. Almost all existing studies about tummy time focused on the relationship between tummy time and developmental milestones in infant. A systematic review found that infants who did tummy time when awake achieved developmental milestones significantly earlier than those who did not or who did limited tummy time when awake in the first 6 months of life [30]. Two other studies also found that tummy time is associated with certain motor milestones achieved during early life [31, 32]. However, whether tummy time has a long term impact on children’s physical activity has not been studied yet. The findings from the present study suggested that promoting tummy time during infancy may lead to increased physical activity in young children. It might be because mothers who practise tummy time earlier and more frequently are more likely to support and encourage their children to play more actively.

Analogous with previous systematic reviews [16, 17], the present study also found boys were more physically active than girls. Children with Australia born mothers played more time outdoors on both weekdays and weekend days. These findings are also important because it helps to develop interventions for targeted population. Having TV time rules at ages 2, 3.5, and 5 years was associated with more children’s outdoor playtime at age 2 years but not at 3.5 and 5 years. The relationship between TV time rules and children’s outdoor playtime needs to be further explored.

Mothers’ knowledge of child development, belief of being able to provide child activities, and high awareness of childhood obesity were not associated with outdoor play and screen-time in 2- to 5-year-olds after adjusting for confounding factors. It may suggest that intervention should be more focused on improving mothers’ skills, practice and own physical activity and sedentary behaviours not only knowledge.

Several limitations of the study need to be considered when interpreting the findings. First, children’s outdoor play and screen-time was reported by mothers that may be subject to recall bias. Also mothers who were employed and those whose children attended child-care might underestimate or overestimate children’s outdoor play and screen-time on weekdays. In order to remedy this limitation, mothers’ employment status and children’s attendance of child-care at 2, 3.5 and 5 years were adjusted, and predictors of outdoor play and screen-time were examined on weekdays and weekend days separately. Second, some important factors were not included in analyses, such as the environment the children were in and mother-child interaction in physical activity. The environment where the children were in may influence the amount of outdoor play and screen-time. However, the environmental variables such as ‘park nearby’, or ‘type of accommodation a child lived in’ were not consistently collected through the 5 year data collection. Therefore, they were not included in analyses. Mother-child interaction in physical activity might be an important factor that promotes young children being physically active. Several previous systematic reviews found that parent-child physical activity interaction and parental encouragement of being physically active were strongly associated with young children’s physical activity level [16, 17, 33]. A recent study showed that the time spent being physically active with their mother at 9-months predicted children’s physical activity at 19-months of age [34]. Third, the study was conducted in South West Sydney, Australia, an area with a relatively low socio-economic level which could limit the generalizability of the study. In addition, this study used data from the Healthy Beginnings trial which was not designed for this purpose and potential bias could be introduced. To address this limitation, we included the intervention allocation in model building.

## Conclusion

Young children’s screen-time was significantly influenced by their mothers’ screen-time and parenting practice on screen viewing. Also early exposure to screen devices could be associated with children’s later screen-time. The early introduction and frequency of tummy time, and informing mothers the importance of playing with their young children could be foundations for children’s future physical activity. Early interventions in improving young children’s physical activity and screen-time should focus on improving pregnant women’s physical activity, awareness of the benefits of playing with their child, reducing their own screen-time as well as providing their children with regular periods of daily tummy time after giving birth.

## Abbreviations

95 % CI: 95 % confidence interval
AOR: Adjusted odds ratio
HBT: Healthy Beginnings Trial.
